# Umbilical Cord Blood Cells Do Not Reduce Ventilation-Induced Lung Injury in Preterm Lambs

**DOI:** 10.3389/fphys.2020.00119

**Published:** 2020-02-21

**Authors:** Madeleine J. Smith, Kyra Y. Y. Chan, Paris Papagianis, Ilias Nitsos, Valerie Zahra, Beth Allison, Graeme R. Polglase, Courtney A. McDonald

**Affiliations:** ^1^The Ritchie Centre, Department of Obstetrics and Gynaecology, Hudson Institute of Medical Research, Monash University, Melbourne, VIC, Australia; ^2^Chronic Infectious and Inflammatory Diseases Research, School of Health and Biomedical Sciences, RMIT University, Bundoora, VIC, Australia

**Keywords:** lung, ventilation-induced lung injury, bronchopulmonary dysplasia, preterm, umbilical cord blood cells, inflammation, stem cells

## Abstract

**Background:**

Preterm infants often have immature lungs and, consequently, many require respiratory support at birth. However, respiratory support causes lung inflammation and injury, termed ventilation-induced lung injury (VILI). Umbilical cord blood (UCB) contains five cell types that have been shown to reduce inflammation and injury. The aim of this study was to determine whether UCB cells can reduce VILI in preterm lambs.

**Methods:**

We assessed lung inflammation and injury, with and without UCB cell administration. Fetal lambs at 125 ± 1 days gestation underwent sterile surgery and were randomly allocated to one of four groups; unoperated controls (UNOP), sham controls (SHAM), injuriously ventilated lambs (VILI), and injuriously ventilated lambs that received UCB cells via the jugular vein 1 h after ventilation (VILI_CELLS_). Ventilated lambs received an injurious ventilation strategy for 15 min, before they were returned to the uterus and the lamb and ewe recovered for 24 h. After 24 h, lambs were delivered via caesarean section and euthanized and the lungs were collected for histological and molecular assessment of inflammation and injury.

**Results:**

VILI led to increased immune cell infiltration, increased cellular proliferation, increased tissue wall thickness, and significantly reduced alveolar septation compared to controls. Further, extracellular matrix proteins collagen and elastin had abnormal deposition following VILI compared to control groups. Administration of UCB cells did not reduce any of these indices.

**Conclusion:**

Administration of UCB cells 1 h after ventilation onset did not reduce VILI in preterm lambs.

## Introduction

Preterm birth is associated with increased risk of infant morbidity and mortality (Blencowe et al., 2012), with a major contributor being lung disease (Moss, 2006). Preterm infants have structurally and functionally immature lungs and therefore, the majority of preterm infants <32 weeks gestational age (GA) require respiratory support at birth (Sharon, 2016). While life-saving, the initiation of respiratory support at birth can cause lung inflammation and injury, particularly if excessive tidal volumes (V_T_) are used (Hillman et al., 2011). Although respiratory support is well-controlled in the Neonatal Intensive Care Unit (NICU), a recent study showed that up to 85% of infants requiring respiratory support in the delivery room received higher V_T_ than recommended (Schmölzer et al., 2010). Indeed, as few as 6 high V_T_ breathes are enough to initiate lung inflammation and injury, known as ventilation-induced lung injury (VILI) (Jobe et al., 2008; Hillman et al., 2010). This detrimental lung injury caused by excess V_T_ in the delivery room may contribute to the progression to chronic lung disease (bronchopulmonary dysplasia) with lifelong consequences (Doyle et al., 2017).

Bronchopumonary dysplasia (BPD) is a severe lung disease, with the main risk factors being preterm birth and mechanical ventilation for 7 or more days. Additionally, there is a correlation between chorioamnionitis and BPD (Jobe, 2011). Despite significant improvements in the care of preterm infants, Doyle et al. (2017) has shown that BPD rates are increasing. BPD is associated with additional adverse outcomes including pulmonary hypertension, cardiovascular disease, and adverse neurological outcomes (Kinsella et al., 2006). VILI is a major antecedent of BPD (Jobe, 2011). VILI is caused by mechanisms including barotrauma volutrauma and atelectrauma, but the main component is believed to be volutrauma (Dreyfuss and Saumon, 1992). The initiation of respiratory support with high tidal volumes leads to lung inflammation and injury via an upregulation of pro-inflammatory cytokines and injury response genes (Jobe et al., 2008; Hillman et al., 2011). There are currently no effective therapies to treat or prevent VILI but targeting the pro-inflammatory cascade may be an effective target for reducing or preventing VILI.

There is increasing interest in the therapeutic potential of stem cells in the reduction of inflammation and injury, in particular lung injury. One promising source of stem cells is umbilical cord blood (UCB), which is usually discarded at birth and is an abundant source of stem and progenitor cells. Studies using both UCB mononuclear cells and individual cell types found within UCB have shown promising results in the attenuation of lung injury following hyperoxia (Mills et al., 2017; Monz et al., 2013). Further, individual UCB cell types have reduced lung injury in various experimental models including mesenchymal stem cells (MSCs) (Chang et al., 2009), T regulatory cells (Tregs) (Garibaldi et al., 2013), and hematopoietic stem cells (De Paepe et al., 2011; Huang et al., 2014). However, there is a lack of evidence to the effectiveness of UCB using large animal models of VILI. In addition, previous studies have administered cell therapies in the days following lung injury, allowing inflammation and tissue remodeling to be well established before treatment. To date, no studies have assessed the therapeutic potential of early or prophylactic UCB within the first hour after birth to reduce VILI.

In this study we assessed whether administration of UCB 1 h after 15 min of injurious ventilation (volutrauma) can reduce lung inflammation and remodeling 24 h later. We hypothesized that UCB cells would reduce histological and molecular indices of lung inflammation and injury when administered intravenously 1 h following volutrauma in preterm lambs.

## Materials and Methods

### Ethics

All animal experimental protocols were approved by the Monash Medical Centre Animal Ethics Committee. All methods were carried out in accordance with the relevant guidelines and regulations as described by the National Health and Medical Research Council of Australia.

### Experimental Design

Sterile fetal surgery was conducted on pregnant ewes at 125 ± 1 (SD) days gestation (term ∼148 days GA). Lambs were randomized to one of four groups; unoperated controls (UNOP) (*n* = 7), sham surgical controls (*n* = 5), injuriously ventilated (*n* = 7: VILI), and injuriously ventilated with UCB cells (*n* = 7: VILI_CELLS_). The experimental protocol spanned 24 h and was conducted at the Monash Biomedical Imaging facility. All UCB-treated animals received cells via intravenous route through the jugular vein catheter. This route was chosen as the cells were given 1 h after injury when ventilation had ceased, intratracheal tubes had been removed and the fetuses had been returned to the uterus.

### Animal Surgery

Pregnant ewes were anesthetized by intravenous injection of thiopentone sodium (20 mg/kg, i.v.; Jurox, NSW, Australia) and were subsequently intubated and anesthesia maintained by inhalational isoflurane (1.5–2.5% in oxygen; Bomac Animal Health, NSW, Australia) delivered via positive pressure ventilation. Polyvinyl catheters (inner diameter 0.86 mm, outer diameter 1.52 mm; Dural Plastics & Engineering, NSW, Australia) were inserted into the left carotid artery and jugular vein of the fetus to measure blood pressure and obtain blood samples and to administer UCB cells, respectively. An ultrasonic flow probe (3PS; Transonic Systems, Ithaca, NY, United States) was placed around the right carotid artery to record carotid blood flow – a pseudo measure of cerebral blood flow (Rudolph, 1983). The incision was sutured using polyvinyl silk. The fetus’s chest was exteriorized, the fetus was dried, and intubated with a 4.5 mm cuffed endotracheal tube and lung liquid was passively drained. SHAM animals remained intubated for 15 min while VILI and VILI_CELLS_ lambs were ventilated using a ventilation strategy aiming to cause volutrauma (Babylog 8000+; Dräger, Lübeck, Germany) by targeting 10–15 ml/kg for a total of 15 min as described previously (Hillman et al., 2007; Barton et al., 2015). Following ventilation, fetuses were returned to the uterus, the incisions closed, and the fetus and ewes recovered. At 1 h post-ventilation, 80 million ovine UCB cells suspended in 3 ml of phosphate-buffered saline were administered to the fetus via the fetal jugular vein catheter (see below). The catheter was flushed with 3 ml of sterile heparinized saline to ensure all the cells were administered. Ewes were monitored overnight and regular blood gases were taken to ensure fetal well-being.

Twenty-four hours after the surgery, ewes were anesthetized as above and the lamb was exteriorized through the same incision site. The lamb was dried, intubated, and ventilated (Vt 7 ml/kg PEEP 5 cm H_2_O) for ∼5 min prior to umbilical cord clamping. A transcutaneous oximeter (Masimo, CA, United States) was attached to its tail. FiO_2_ was initially set at 0.4 and adjusted to maintain SaO_2_ between 88 and 95%. Intratracheal surfactant (240 mg in 3 ml; Curosurf^®^; Chiesi Pharmaceuticals, Parma, Italy) was administered within the first 10 min to improve lung compliance and sustained inflations were applied to recruit the lung if necessary. For unoperated control (UNOP) lambs, the ewe and lambs underwent the surgery, catheterization, and ventilation as described above.

Once the lamb was stabilized, the umbilical cord was clamped and the lamb was transferred to an MRI-compatible ventilator (Pneupac babyPAC^TM^; Pneupac, Smiths Medical, United Kingdom). Lambs were scanned for 60 min in a 3T MRI scanner to asses brain structure, spectroscopy, and diffusion imaging. At the end of scanning lambs were euthanized with an overdose of sodium pentobarbitone (>100 mg/kg, i.v. Valabarb Euthanasia Solution; Jurox, NSW, Australia). Ewes were similarly euthanized immediately after delivery of the lamb.

### Umbilical Cord Blood Cell Preparation

Ovine allogeneic term (141 days gestation) UCB sample was obtained from a separate group of animals as previously described (Aridas et al., 2016). Briefly, after clamping of the cord, UCB was collected via the two umbilical veins and approximately 90 ml of blood was collected from each ovine fetus. The mononuclear layer of cells was obtained by centrifuging the blood at 1000 × *g* for 12 min with no brake and cells were suspended in phosphate-buffered saline (PBS; Gibco, Waltham, MA, United States). Red blood cells were lysed using red blood cell lysis buffer (155 nM ammonium chloride, 10 mM potassium bicarbonate, and 0.1 mM EDTA in MilliQ water). The lysis reaction was stopped with excess media [16.5% fetal bovine serum (FBS) in DMEM:F13, Gibco, Waltham, MA, United States]. Cells were manually counted and their viability was assessed using trypan blue exclusion dye (Gibco, Waltham, MA, United States) and a hemocytometer. UCB cells were cryopreserved in dimethyl sulfoxide (10% DMSO, Sigma in FBS) and stored in liquid nitrogen for subsequent administration to lambs. UCB cells were thawed as previously described and have shown that administration of immediately thawed cells is effective at reducing inflammation and cell death (Aridas et al., 2016; Li et al., 2017; Paton et al., 2018; Paton et al., 2019). Briefly, cells were rapidly thawed in a 37°C water bath with 10 ml media, centrifuged for 5 min at 300 × *g* and then supernatant was removed and cells resuspended in media and counted using a hemocytometer and trypan blue. Cells were then left on ice until administration.

### Histological Analysis

At postmortem the lungs were dissected from the chest and the right upper lobe was pressure fixed at 20 cm H_2_O by inflation with 10% neutral buffered formalin (pH 7.4) for histological examination. The lobe was cut into 5 mm slices and cut into 2 cm^2^ sections and three sections of lung were randomly chosen and embedded in paraffin. Five micrometers of sections were stained and a total of 15 random high-power images were taken for each stain. Sections were stained with Hematoxylin and Eosin (H&E), Hart’s resorcin-fuchsin stain to identify elastin or picrosirius red to stain for type I and III collagen. H&E sections were scored for alveolar wall thickness, as described previously (Hillman et al., 2010; Polglase et al., 2017). Elastin and collagen density was measured by image analysis (ImagePro Plus) using five random fields of view per section using a 40× objective lens. Staining density was adjusted for tissue area. Analysis was performed using Image Pro Premier software. The total area of collagen/elastin within each image was calculated and normalized to the area of tissue. To determine the number of secondary septal crests present in the HART’s stained sections, the image processing package Fiji was used, with a grid overlay with 1131 dots was placed over each image. The number of dots landing on tissue, airspace, and secondary septal crests were counted. The number of secondary septal crests was divided by the area of tissue and multiplied by 100 to yield a secondary septal crest percentage.

### Immunohistochemical Analysis

Proliferating cells were visualized using Ki67 (1:200, Thermo Fisher Scientific, Cat#MA5-14520) immunohistochemistry. Immune cells were visualized using CD45 (1:500, AbCam, Cat#ab10558). Stained lung sections were analyzed in duplicate images, with each lung section imaged across five fields of view per slide. Briefly, for each immunohistochemical stain, lung sections were dewaxed through xylene–alcohol series. Antigen retrieval was performed (Ki67; in 0.1 M citrate buffer, CD45; DAKO PT Link in 1× DAKO Target Retrieval Solution). Sections were then blocked (Ki67; 5% BSA in 1% triton X PBS, CD45; DAKO Protein Block). All sections were incubated with primary antibody overnight at 4°. The sections were then incubated in secondary antibody (Ki67; 1:200 biotinylated goat anti-rabbit IgG antibody, Thermo Scientific, CD45; 1:500, Dako) for 1 h. Staining was revealed with 3,3′-diaminobenzidine (Pierce Biotechnology, Rockford, IL, United States). Ki67 sections were counterstained with Harris hemotoxylin. All slides were then cleared and cover-slipped using mounting medium (DPX, Merck, Kilsyth, Australia). Slides were dried then visualized using light microscopy (Ki67; Olympus Microscope, Japan, CD45; Leica ScanScope AT Turbo and captured using Leica Scanner Console version 102.7.5). Cell counts were performed using Image J (NIH, Bethesda, MD, United States) at 400× magnification.

### Gene Analysis

The lower lobe of the right lung was chopped into small pieces and immediately snap-frozen in liquid nitrogen. Twenty milligrams of lung tissue was homogenized and total RNA isolated (RNeasy Midi Extraction Kit, Qiagen, Australia) and reserve-transcribed into cDNA (SuperScript III reverse transcriptase; Invitrogen). Genes of interest were measured by qPCR/Taqman. Five microliters of cDNA from each sample was plated onto a 96-well PCR plate and submitted to the MHTP Genomics Facility, Hudson Institute of Medical Research. Quality control testing was performed using Sybr chemistry for the housekeeping gene 18s (ABI 7900 HT qPCR). This was followed by preamplification, then taqman analysis of the seven genes listed in Table 1. Relative mRNA levels of gene expression are expressed relative to the mean of the UNOP group.

**TABLE 1:** List of ovine specific probes for TaqMan Gene Array.

**Gene/probe**	**Assay ID**
18s (housekeeping gene)	Oa4906333_g1
IL-6	Oa04656315_m1
IL-8	Bt03211906_m1
IL-10	Oa03212724_m1
IL-1β	Oa04656322_m1
CTGF	Oa04659069_g1
CYR61	Oa04673852_g1
EGR1	Oa03237885_m1

### Statistical Analysis

Fetal parameters were compared using one-way ANOVA. Ventilation parameters were compared using two-way repeated measures ANOVA and Holm–Sidak *post hoc* analysis, using SigmaStat. Indices of lung inflammation and injury were analyzed by using GraphPad Prism 7. Data were analyzed using one-way ANOVA or Kruskal–Wallis, as appropriate, with Tukey’ or Dunn’s multiple comparisons test, respectively. Data are expressed as mean ± standard error of the mean (SEM). Statistical significance was considered as *p* < 0.05.

## Results

### Fetal Physiological Parameters

Fetal parameters are summarized in Table 2. There were more males in each group compared to females but no difference between groups was observed. Birth weight and lung weights were not different between groups. There was no difference in fetal blood gas parameters, pH, PaCO_2_, PaO_2_, and SaO_2_ between groups.

**TABLE 2:** Baseline fetal data for all groups recorded prior to ventilation for all groups except UNOP, which was recorded before delivery.

	**UNOP**	**SHAM**	**VILI**	**VILI_CELLS_**
Number	7	5	7	7
Sex (male)	6 (86%)	3 (60%)	5 (71%)	4 (57%)
Birth weight (kg)	3.4 ± 0.2	3.3 ± 0.1	3.4 ± 0.2	3.4 ± 0.2
Lung weight (g)	150.5 ± 33	137.9 ± 46	147.0 ± 41	141.6 ± 37
Birth order 1st (%)	6 (86%)	4 (80%)	7 (100%)	7 (100%)
pH	7.27 ± 0.02	7.27 ± 0.02	7.25 ± 0.02	7.27 ± 0.01
PaO_2_	36.7 ± 7.2	33.0 ± 2.7	51.2 ± 8.8	34.1 ± 1.3
PaCO_2_	59.0 ± 3.2	64.0 ± 5.8	57.3 ± 3.1	59.2 ± 1.6
SaO_2_	48.2 ± 9.4	46.4 ± 5.7	67.5 ± 6.6	49.0 ± 2.9

*Data are expressed as mean ± SD.*

### Ventilation

**FIGURE 1:**
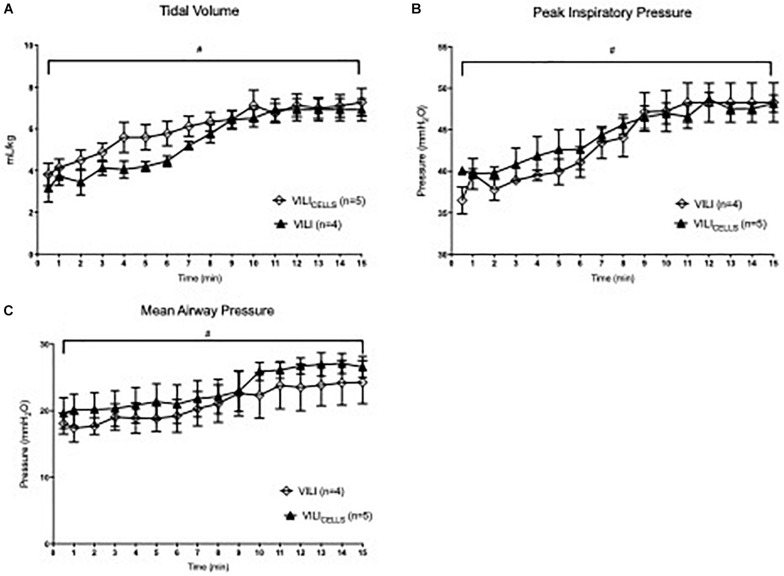
Ventilation parameters for the two ventilated groups; VILI and VILI_CELLS_. **(A)** Tidal volume, **(B)** peak inspiratory pressure, and **(C)** mean airway pressure. # indicates that time had a significant effect on the given ventilation parameter (^#^*p* < 0.05).

Ventilation parameters (Figure 1) during the volutrauma, including PIP, V_T_, and mean airway pressure were not different between VILI and VILI_CELLS_. V_T_ significantly increased with time throughout the 15 min ventilation strategy in both groups reaching a peak of 8.1 and 8.4 ml/kg for the VILI and VILI_CELLS_ group, respectively (*P* < 0.001 for both groups).

### Blood Gas Parameters

**FIGURE 2:**
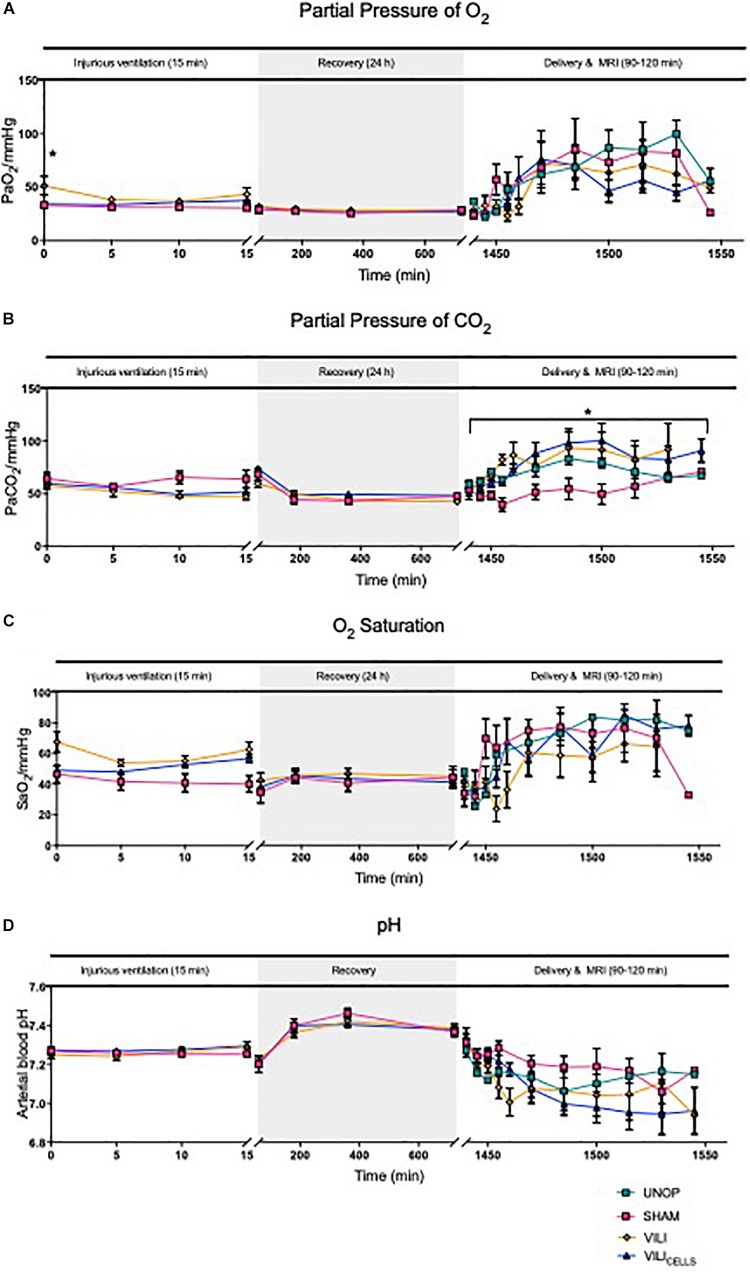
Blood gas parameters between SHAM, VILI, and VILI_CELLS_ for the injurious ventilation period and recovery period and between UNOP, SHAM, VILI, and VILI_CELLS_ for delivery and MRI; partial pressure of **(A)** O_2_, **(B)** CO_2_, **(C)** saturation of oxygen, and **(D)** pH. Asterisk in panel **(A)** indicates significant difference in PaO_2_ at time 0 between VILI and SHAM groups. Asterisk in panel **(B)** indicates difference in PaCO_2_ during delivery and MRI, with SHAM significantly lower than VILI and VILI_CELLS_ groups, *P < 0.05.

Partial pressure of oxygen (PaO_2_), partial pressure of carbon dioxide (PaCO_2_), arterial saturation of oxygen (SaO_2_), and pH were measured regularly. PaO_2_ was significantly higher in the VILI group at the onset of ventilation compared to the SHAM group (*P* = 0.017; Figure 2A). During recovery there was no significant difference between groups. At 24 h during the MRI, PaCO_2_ was significantly lower in the SHAM group compared to VILI (*P* = 0.04) and VILI_CELLS_ (*P* = 0.036) lambs (Figure 2B). There was no significant different in SaO_2_ (Figure 2C) or pH (Figure 2D) across the experiment.

### Assessment of Lung Injury

For all molecular and histological measures of lung inflammation and injury there was no significant difference between the UNOP and SHAM group; therefore, these groups were pooled (Controls).

**FIGURE 3:**
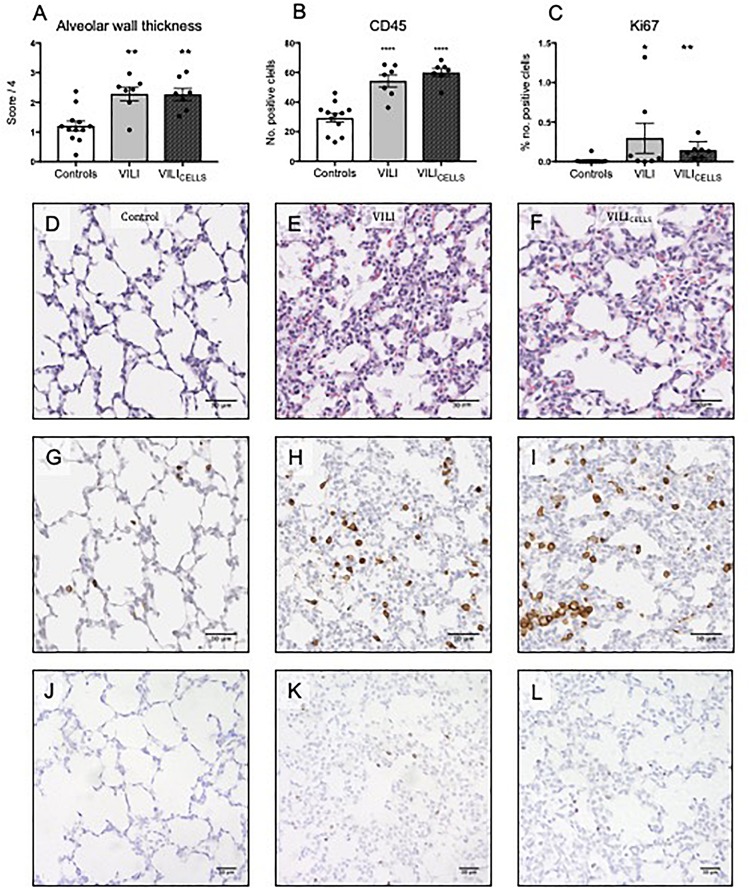
**(A)** Alveolar wall thickness expressed a score out of 4, **(B)** Immune cell infiltration expressed as the number of positive cells per field of view, **(C)** number of proliferating cells expressed as the percentage of positive cells. Scale bars show 50 μm. *P < 0.05, **P < 0.01, ****P < 0.0001 compared to controls. **(D–F)** Representative lung sections stained with Hematoxylin and Eosin to assess alveolar wall thickness in **(D)** control, **(E)** VILI, and **(F)** VILI_CELLS_ lambs. **(G–I)** Representative lung sections demonstrating immune cell presence using CD45 immunohistochemistry. **(J–L)** Representative lung sections demonstrating cellular proliferation using Ki67 immunohistochemistry. Scale bar shows 30 μm.

Alveolar wall thickness (Figures 3A,D–F), inflammatory cell infiltration (Figures 3B,G–I), and proliferating cells (Figures 3C,J–L) were significantly increased in both the VILI (*P* = 0.0017, *P* < 0.0001, and *P* = 0.0367, respectively) and VILI_CELLS_ (*P* = 0.002, *P* < 0.0001, and *P* = 0.0014, respectively) groups compared to controls. There was no difference in these parameters between VILI and VILI_CELLS_. There was no difference in hyaline membranes, epithelial sloughing, and hemorrhage between the groups.

### Assessment of Lung Structure of Composition

**FIGURE 4:**
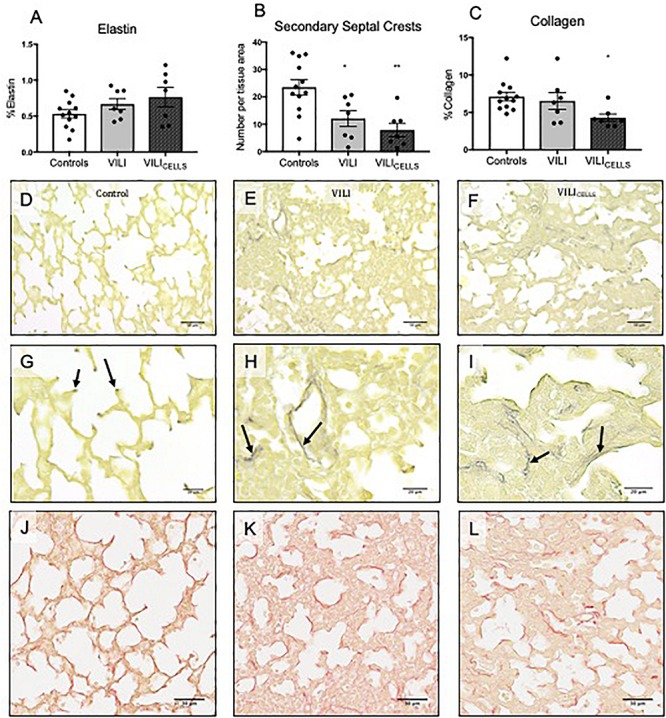
**(A)** %Elastin content in tissue. **(B)** Density of secondary septal crests expressed as number per tissue area. **(C)** %Collagen content in tissue. *P < 0.05, **P < 0.01 compared to controls. **(D–L)** Representative lung sections stained with HARTs to view elastin (black staining) in **(D,G)** control **(E,H)** VILI, and **(F,I)** VILI_CELLS_. Arrows indicate elastin deposition at the points of septa in control lambs, which is irregular in the VILI and VILI_CELLS_ lambs. **(J–L)** Representative lung sections stained with picrosirius red to view collagen (dark red staining).

Quantitative assessment of elastin showed no difference between groups for the percentage of elastin in tissue (Figures 4A,D–I). The number of secondary septal crests was significantly decreased in the VILI and VILI_CELLS_ groups compared to controls (*P* = 0.0247 and *P* = 0.0021, respectively, Figures 4B,J–L). Qualitative observation showed that elastin fibers were more elongated and relocated to airway walls in the ventilated (VILI and VILI_CELLS_) groups (Figures 4G–I) rather than being localized to the septal crests as observed in control lungs.

Quantitative assessment of collagen showed that the VILI_CELLS_ group had significantly less collagen in tissue compared to the control group (*P* = 0.0302, Figure 4C). There was no significant difference in collagen between the VILI and control groups. Again, qualitative observation showed that collagen fibers were more elongated in the ventilated (VILI and VILI_CELLS_) groups (Figures 4K,L).

### Assessment of Gene Expression

**FIGURE 5:**
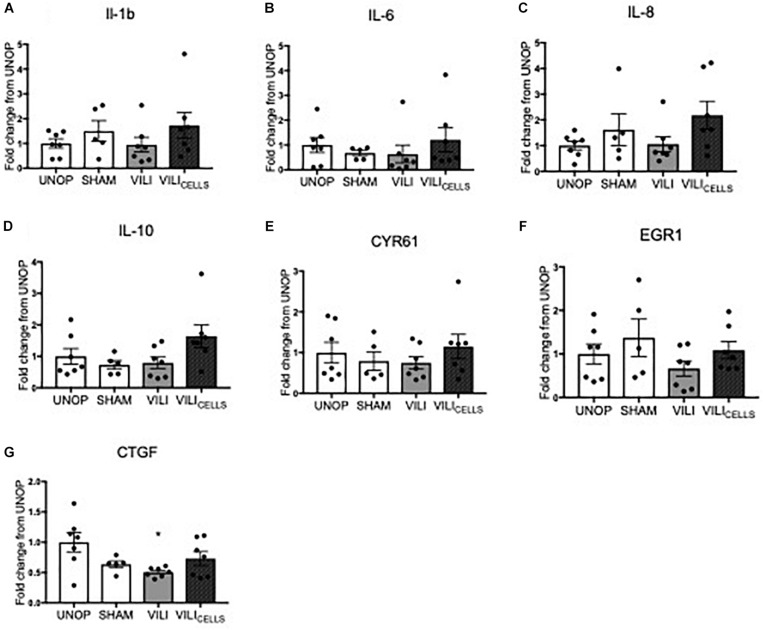
Relative fold increase of **(A)** interleukin (IL)-1β, **(B)** IL-6, **(C)** IL-8, **(D)** IL-10, **(E)** cysteine rich 61 (CYR61), **(F)** early growth response 1 (EGR1), **(G)** connective tissue growth factor (CTGF) expression between treatment groups expressed as a fold increase from UNOP.

mRNA levels of proinflammatory cytokines interleukin (IL)-1β, IL-6, IL-8, and anti-inflammatory cytokine IL-10 and early response genes (CYR61, EGR1, and CTGF) were not different between groups as seen in Figure 5.

## Discussion

Preterm infants that require resuscitation in the delivery room may inadvertently receive higher tidal volumes than recommended, resulting in VILI, a major cause of BPD (Schmölzer et al., 2010). Similar to previous studies (Hillman et al., 2007), 15 min of volutrauma caused lung inflammation and injury and profound changes to lung structure. UCB cells have inherent properties which we hypothesized would reduce lung inflammation and injury. However, our study showed that the administration of UCB cells, 1 h after volutrauma, did not reduce lung inflammation or injury suggesting that early administration of UCB cells may not be efficacious at reducing or preventing VILI.

We investigated UCB cells because previous studies using UCB cells have demonstrated their anti-inflammatory and anti-fibrotic properties and their ability to aid alveolar epithelial reconstitution (Chang et al., 2009; De Paepe et al., 2011; Garibaldi et al., 2013; Monz et al., 2013; Huang et al., 2014; Mills et al., 2017). In our study UCB cells were unable to reduce the increase in alveolar wall thickness and the number of infiltrating immune cells or prevent the loss of secondary septal crests from VILI. The mononuclear cell layer from UCB blood was chosen in this study over individual cell types. A study from our group has directly compared individual cell types and the UCB mononuclear cell fraction, showing that both reduced deficits and inflammation following brain injury (McDonald et al., 2018). Further, a large number of cells was required for this model and to use individual cell types, several cord blood units would be required. In the future, when cell expansion technologies are more advanced and larger doses of individual UCB stem cells can be generated, it would be interesting to examine different cell types in a similar experiment. UCB cells have never been studied for the treatment of VILI in a large animal model, so our chosen dose was based on previous studies treating brain injury in fetal sheep. In these studies, dosages of 25 million UCB cells/kg were able to attenuate hypoxic ischemic term and preterm brain injury and showed that both autologous and allogeneic UCB cell administration were effective at reducing inflammation and cell death (Aridas et al., 2016; Li et al., 2016). Although studies have not used UCB cells for lung injury in fetal sheep, the same dose of hAECs alleviates both brain and lung injury in fetal sheep (Vosdoganes et al., 2011; Yawno et al., 2013). Our dose was based on an estimate of 3.2 kg fetal weight. Our average birth weight of 3.4 kg meant an average dose of ∼23.5 million cells/kg was delivered. It is unlikely the marginally lower dose delivered in this study would have altered the findings. However, as this is the first study to assess the effectiveness of UCB cells in a large animal model of lung injury, it is likely that future studies will need to assess different doses. The UCB cells used in this study were from sheep UCB, due to the limited availability of sheep-specific antibodies, we were unable to identify the proportion of various cell types within the UCB samples. However, previous studies have shown UCB cells from autologous and allogeneic sheep to be efficacious in a sheep model of preterm brain injury (Li et al., 2017).

Umbilical cord blood cells were administered 1 h after volutrauma, which is known to be the peak of the pulmonary and systemic pro-inflammatory cascade resultant from injurious respiratory support (Hillman et al., 2011). This time point for stem cell administration has not been explored before. It was our primary goal to see whether UCBs given at this peak inflammatory time could dampen the inflammatory response, thus reducing or preventing the subsequent progression of lung injury. Given the lack of efficacy, it is important to consider whether introducing the cells into a profound pro-inflammatory environment could alter the efficacy of UCB cells. Although the majority of studies that use cells such as MSCs, a stem cell subset present in UCB, have shown reduced inflammation (Curley et al., 2012; Hayes et al., 2015; Lai et al., 2015), there are some studies that have shown that MSCs can exacerbate the inflammatory response if incorporated into an established pro-inflammatory environment (Bernardo and Fibbe, 2013). It has also been shown that bone marrow-derived MSCs are able to stimulate mononuclear cell types to produce pro-inflammatory cytokines IL-6 and IFN-γ (Rasmusson et al., 2007). Although we did not see any differences in inflammation at 24 h, it is likely that we missed the cytokine increases given they are largely resolved at this time (Hillman et al., 2011). Importantly, our results indicate that the UCB cells are not effective during this peak inflammatory period. Previous studies have administered human amnion epithelial cells prior to the onset of ventilation (Melville et al., 2017), 3 and 6 h (Hodges et al., 2012) into ventilation. Further, previous studies administered MSCs prior to the onset of ventilation (Chimenti et al., 2012) or 14 days after lung hyperoxia (Hansmann et al., 2012). This is the first study to assess cell administration at 1 h following lung injury, suggesting that cell administration may be efficacious in the resolution or prevention of inflammation and injury may not be efficacious when given at the peak of the pro-inflammatory cascade. Future studies may be necessary to avoid this time point by administering cells prophylactically at the onset of ventilation or 6 h after the cessation of ventilation.

We used an exteriorized model of VILI to test the UCBs as described previously by others (Hillman et al., 2011). This model was chosen due to its ability to isolate the initial volutrauma from subsequent inflammation and injury that would occur over the proceeding 24 h with maintaining these lambs *ex utero*, including the need for ongoing respiratory support, nutrition, and neonatal intensive care. Instead, the ewe (via the placenta) provides all of the respiratory and nutritional requirements of the fetus. One potential problem with this model is that the presence of a fetal circulation, which means that only 12% of combined ventricular output passes through the pulmonary circulation (Rudolph, 1983). Normally when cells are given intravenously after birth, they can become passively trapped within the lungs, for a few days/weeks after administration (Anjos-Afonso et al., 2004; Kean et al., 2013). It has been postulated that this may actually be one of the mechanisms where by stem cells are effective for reducing lung injury (Inamdar and Inamdar, 2013). However, it is known that actual engraftment and differentiation of UCB cells occurs at very low rates, or not at all, at the site of injury (Paton et al., 2018). Instead, they act systemically to reduce inflammation and can act at immune organs such as the spleen and lymph nodes (McDonald et al., 2018). In this study we did not label the UCB cells so we were unable to determine if there was cell engraftment within the lung parenchyma. However, studies by Vosdoganes et al. (2011) showed that i.v. administration of hAECs to the fetus reduced the pulmonary inflammatory response in an LPS model of lung injury. Although in this study combined intratracheal and i.v. administration was most effective, both administration routes alone were able to attenuate injury. A study by Cargnoni et al. (2009) using a bleomycin-induced lung injury mouse model showed that both i.v. and intratracheal hAEC administration was able to attenuate lung injury. The optimal route of cell administration needs to be investigated further.

While UCB cell administration did not reduce VILI in this study, the potential of UCB administration should not be dismissed out of hand. A multiple dosing scheme may be more effective at reducing inflammation and injury, since UCB cells unexposed to inflammation will be given at multiple time points, potentially overcoming a loss of efficacy following inflammation exposure. UCB cell administration at 1 h following initiation of ventilation is ineffective; therefore, a study comparing other or even multiple time points may show benefit.

## Conclusion

We demonstrated that an acute period of volutrauma resulted in profound changes to lung architecture that can be observed 24 h after the insult, confirming the importance of the initial resuscitation period in the delivery room to the progression and severity of VILI. This highlights the importance of better monitoring and/or control of tidal volume during the initial resuscitation of preterm infants, and also the importance of this critical window for therapeutic targeting. UCB cells administered 1 h after volutrauma were unable to reduce VILI in preterm fetal sheep. Future studies will be needed to determine the optimal timing, dosing, or administration scheme before UCB cells can be seen as a potential prophylactic therapy for VILI in preterm infants.

## Data Availability Statement

The datasets generated for this study are available on request to the corresponding author.

## Ethics Statement

The animal study was reviewed and approved by the Monash University Animal Ethics Committee.

## Author Contributions

MS, KC, PP, IN, GP, and CM had input into the conception and design of the work. MS, KC, PP, VZ, IN, BA, GP, and CM were involved with data acquisition, analysis and interpretation of data for the work, drafting and revising the work, and approved the content for publication.

## Conflict of Interest

The authors declare that the research was conducted in the absence of any commercial or financial relationships that could be construed as a potential conflict of interest.
